# A Novel Large Duplication on the X Chromosome as a Cause of Familial Generalized Dystonia: A Case Report

**DOI:** 10.3390/ijms26020809

**Published:** 2025-01-19

**Authors:** António Costa, Diogo Pereira, Maria João Malaquias, Ana Filipa Brandão, Jorge Oliveira, Marina Magalhães

**Affiliations:** 1 Department of Neurology, Centro Hospitalar Universitário de Santo António, Unidade Local de Saúde de Santo António, 4099-001 Porto, Portugal; diogoteixeira.neurologia@chporto.min-saude.pt (D.P.); marinamagalhaes.neurologia@chporto.min-saude.pt (M.M.); 2 Department of Neurology, Unidade Local de Saúde de Gaia/Espinho, 4434-502 Porto, Portugal; maria.malaquias@ulsge.min-saude.pt; 3 CGPP—Centro de Genética Preditiva e Preventiva, IBMC—Instituto de Biologia Molecular e Celular, Universidade do Porto, 4200-135 Porto, Portugal; ana.brandao@ibmc.up.pt (A.F.B.); jmoliveira@ibmc.up.pt (J.O.); 4 i3S—Instituto de Investigação e Inovação em Saúde, Universidade do Porto, 4200-135 Porto, Portugal; 5 Laboratory of Cell Biology, Department of Microscopy, ICBAS—Instituto de Ciências Biomédicas Abel Salazar, Universidade do Porto, 4050-313 Porto, Portugal; 6 UMIB—Unit for Multidisciplinary Research in Biomedicine, ICBAS/ITR-Laboratory for Integrative and Translational Research in Population Health, Universidade do Porto, 4050-313 Porto, Portugal

**Keywords:** chromosomopathy, generalized dystonia, chromosome inactivation

## Abstract

Chromosomal aberrations are rare but known causes of movement disorders, presenting with broad phenotypes in which dystonia may be predominant. During the investigation of such cases, chromosomal studies are not often considered as a first approach. In this article, the authors describe a family affected by a generalized form of dystonia, evolving from a focal phenotype, for which a new X chromosome large duplication was found to be the likely causative, therefore highlighting the role of such studies when facing complex movement disorders.

## 1. Introduction

Chromosomopathies can cause various movement disorders [1]. While these occur quite rarely, they should be considered in the pediatric population, as reported data suggest that in such populations, these abnormalities may justify movement disorders of suspected genetic etiology in up to 28% of cases [2]. 

Movement disorders associated with chromosomal abnormalities may present as tremor and parkinsonism, commonly attributed to aneuploidies of sex chromosomes [3,4], ataxia [1], and dystonia [5]. A recent systematic review suggested that aneuploidies of sex chromosomes are relatively common among patients with tremor and parkinsonism [4], highlighting the importance of considering chromosomal studies, especially in such cases. In the case of genetically caused dystonia, as clearer genotype–phenotype correlations are being established and genetic testing becomes more widely available, the list of monogenic causes continues to expand [6]. As an example of the dystonic phenotype, patients with 18p deletion syndrome exhibit short stature, facial dysmorphism, intellectual disability, developmental delay, and commonly dystonia, although chorea, myoclonus, tremor, tics, and ataxia are also described [5]. Several other dystonic conditions affecting the X-chromosome can also be listed, such as DYT3, where focal to generalized progressive dystonia is followed by parkinsonism [7]; ATR-X syndrome, where patients may develop a myoclonus-dystonia phenotype [8]; and Lesch–Nyhan syndrome [9]. These are monogenic conditions resulting from specific X-linked gene mutations (*TAF1*, *ATRX*, and *HPRT1* genes, respectively). 

Chromosomopathy-derived phenotypes are usually part of a broader clinical picture, including other symptoms such as dysmorphic features, developmental disabilities, and epilepsy. Although chromosomal studies are not typically the first diagnostic tests for complex movement disorders, chromosomal microarray analysis (CMA) has been recommended when there is such a co-occurrence of clinical features [1,10]. Nevertheless, chromosomal aberrations tend to produce more pronounced abnormalities in genome expression, making them more likely to be considered in cases involving obvious neurodevelopmental delays, and potentially overlooked when hypothesizing about specific circumstances of movement disorders. The authors describe the case of a Portuguese family in which extensive genetic investigation identified a novel X chromosome duplication in two first-degree family members. Both presented with generalized dystonia that evolved from childhood-onset focal dystonia. Additional genetic studies supported the classification of the identified variant as likely disease causing.

## 2. Case Report

**Figure 1:**
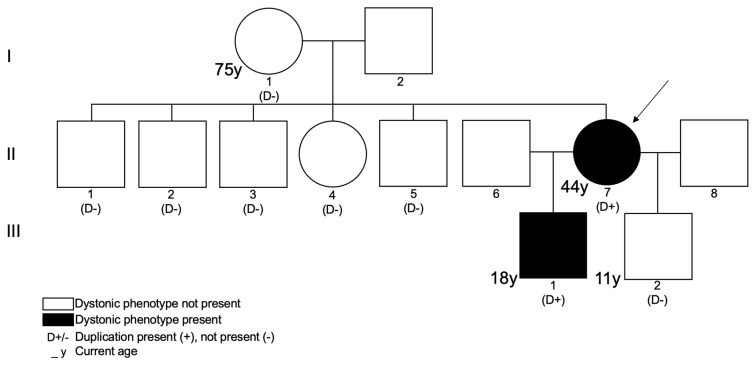
Pedigree of the described family. Index case is represented by II7. Patients III1 and III2 have different, non-consanguineous fathers.

We report the case of a 44-year-old woman (II7 on Figure 1) with a generalized dystonia associated with slight cognitive impairment with learning difficulties and behavior problems. She presented with lower limb dystonia at the age of 12, which slowly progressed to generalized dystonia affecting speech, the cranial–cervical region, and limbs. Irregular hand tremors and brisk reflexes were also noted. The dystonia was always mild and never disabling, but due to cognitive and behavioral problems, this patient has been unable to sustain professional activities over the years, despite multiple attempts at securing employment. This situation necessitated her integration into an institution for individuals with special needs, where occupational therapy could be provided. The patient never presented dysmorphic features. She has been symptomatically treated with levodopa, trihexyphenidyl, and botulinum toxin.

Video S1 (Supplementary File I) presents the evaluation of patient II7 (referred to as P1 in the video) at two timepoints: at age 12, showing left-predominant generalized dystonia during walking and bilateral hand dystonia, more pronounced on the left, when writing; and at age 32, exhibiting laryngeal dystonia and right laterocollis while speaking (in Portuguese).

**Figure 2:**
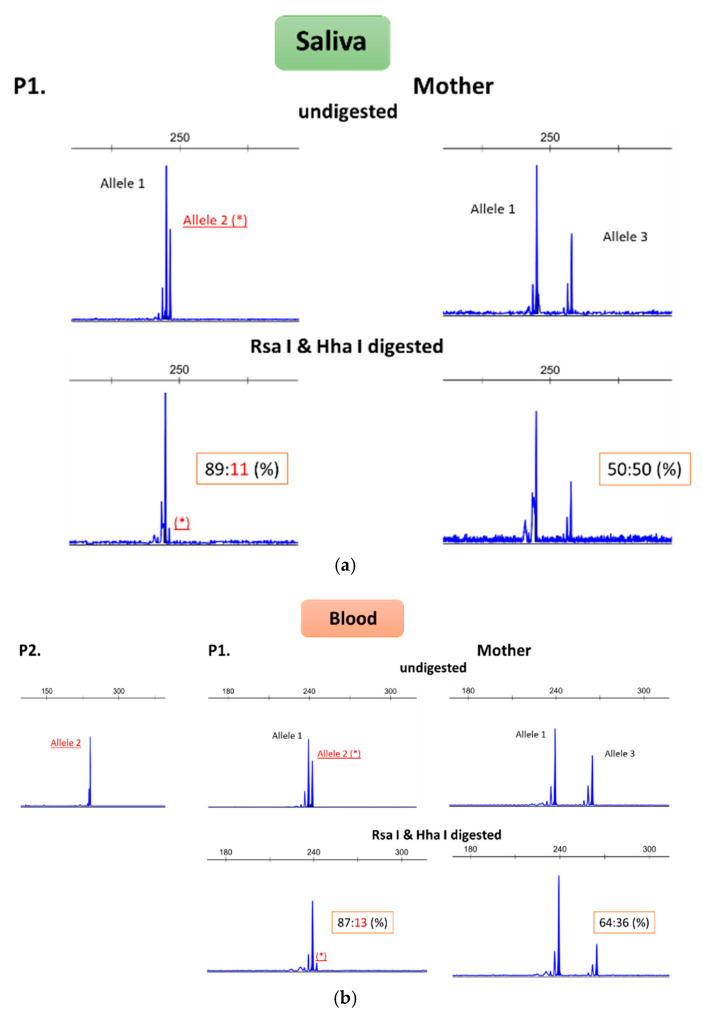
Results of X chromosome inactivation studies in patient II7 (here referenced as P1), her mother, and affected son III1 (here referenced as P2). X chromosome inactivation studies were performed on DNA extracted from saliva (**a**) and blood (**b**). The top figures depict PCR of the HUMARA gene without prior digestion, showing different size alleles. PCR amplification was also performed on DNA previously digested with Rsa I and Hha I enzymes. Effective digestion occurs when the DNA is unmethylated (these enzymes are sensitive to methylation). Therefore, after this step, the peak corresponding to the active allele will be smaller. By comparing the two peak areas, it is possible to determine the percentage of inactivation (ideally it should be 50:50%, indicating X chromosome random inactivation). In patient II7, the mutated chromosome (allele 2) is preferentially activated, whereas in the mother, the pattern suggests random inactivation. * represents the X chromosome allele carrying the duplication, which is not methylated in II7.

The initial etiological investigation included the major neurometabolic conditions associated with dystonia, brain magnetic resonance imaging (MRI), electroencephalography, and electromyography, all of which showed no abnormalities. A next-generation sequencing multigene panel of 150 genes based on whole-exome sequencing (WES) for dystonia was negative (Supplementary Data S1). Clinical exome sequencing was then conducted using previously generated WES data (Supplementary Data S1). An X chromosome duplication was found in II7 at Xq13.3q21.1 [NC_000023.10:g. (74376207_76709547)_(79286710_79932008)dup], involving 14 genes, 7 of which are listed on the Online Mendelian Inheritance in Man (OMIM) Morbid Map (Table 1). Further confirmation with CMA performed on II7 estimated its size to be approximately 4 Mb long (arr[hg19]Xq13.3q21.1(75640831-79709679)x3)). Duplication segregation studies within the family showed that it was absent in II7’s mother and in her five siblings (one sister and four brothers). Parallel analysis using polymorphic short tandem repeats demonstrated that NC_000023.10:g.(74376207_76709547)_(79286710_79932008)dup occurred de novo in II7. Subsequent X chromosome inactivation studies (Figure 2) using the human androgen receptor (HUMARA) assay [11] revealed in II7 a skewed inactivation, suggesting preferential activation of the X chromosome harboring the duplication, a pattern consistent in both blood and saliva DNA samples.

The index patient had two male children (Figure 1), aged 18 (III1) and 11 (III2), from two different non-consanguineous relationships. Both children were delivered without complications and do not present with dysmorphic features. Patient III1 presented since the age of 3 an abnormal posture, which evolved into a slight generalized dystonia associated with speech problems, laxity, and learning difficulties. Video S1 also shows the evaluation of patient III1 (referenced to as P2) at 6 years old, showing a bilateral dystonic posture of the hands and feet, also more pronounced on the left side. Myoclonic epilepsy developed at the age of 15. Brain MRI and electroencephalography were normal.

Patient III2 exhibited some learning difficulties at the age of 6 and presented a particular way of writing and holding cutlery. Aside from these minor particularities, until nowadays, a clear dystonic trait has not been noted.

Variant segregation analysis was performed by quantitative PCR (qPCR) in III1 and III2, confirming the same duplication in III1, but not in III2.

## 3. Discussion

**Figure 3:**
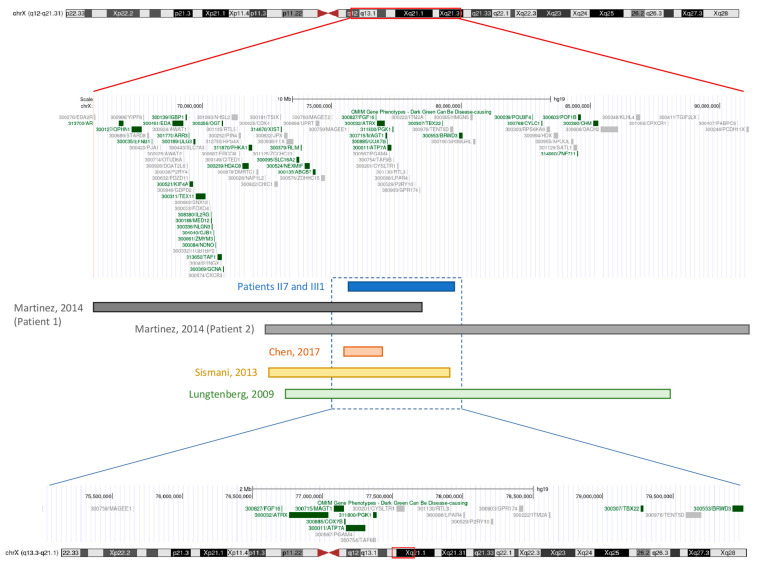
Physical map of the duplicated Xq13.3-q21.1 region in patients II7 and III1 (in blue), along with the duplicated regions involved in previously reported cases, as mentioned in Table 1 (in grey, patients described by Martinez et al. [12]; in orange, patient described by Chen et al. [13]; in yellow, patient described by Sismani et al. [14]; in green, patient described by Lugtenberg et al. [15]; adapted from the UCSC Genome Browser (https://genome.ucsc.edu/ (accessed on 1 October 2024)).

The authors found five cases in the literature with well documented duplications in or near the Xq13.3q21.1 region, manifesting with severe intellectual disability and dysmorphic features, but never with dystonia (Table 1) [12,13,14,15]. A physical map of the duplicated Xq13.3-q21.1 region in this family, along with the duplicated regions involved in published cases, is depicted in Figure 3. Hypotonia was described in two unrelated male children with this duplication [12], as well as in a patient with a duplication on a similar region, Xq13.2-q21.1 [15]. Two reports described fetuses whose gestations were terminated early after prenatal studies identified a similar alteration; therefore, no descriptions of hypotonia or dystonia were provided [13,14]. In comparison to the family described in this report, previously documented cases exhibit notable differences in both neurodevelopmental and movement disorder outcomes. While earlier cases share duplications in regions overlapping or adjacent to Xq13.3q21.1, their primary clinical manifestations include severe intellectual disability, profoundly impairing the attainment of basic neurodevelopmental milestones. Additionally, prominent dysmorphic features were described involving not only neurological structures, but also facial morphology, appendicular and truncal anatomy, as well as internal organs and genitalia. In contrast, the intellectual impairment observed in this study’s family is notably milder, with only mild-to-moderate deficits affecting II7. Furthermore, the absence of significant dysmorphic traits majorly differentiates II7, III1, and III2 from the abovementioned examples. Most strikingly, the dystonic phenotype observed in II7 and III1 emerges as the primary clinical characteristic, representing a key difference from prior reports in which movement disorders, including dystonia, were not described. 

It is important to note that the duplicated regions are not identical across all of these cases. Variations in the specific duplicated genetic material, along with other potential underlying mechanisms, likely contribute to the observed phenotypic differences. The duplicated region contains a total of 22 genes and 18 pseudogenes (https://search.clinicalgenome.org (accessed on 1 October 2024)). All OMIM-reported genes involved in this case have also been identified in partially overlapping duplications reported in other cases (Table 1). For example, in three cases [12,14], all patients shared the affected genes *SLC16A2* and *ZDHHC15*, and in two, *BRWD3* and *ZNF711* were also shared. The duplication found in our patient is classified as pathogenic (Supplementary Data S2—from Varsome software, version: 12.8.2 [16]), but the disease mechanism is yet to be fully characterized. Two plausible explanations could account for the observed phenotypic differences among patients with Xq13.3-q21.1 duplications:

(a) The association with an increase in gene dosage, although this mechanism has not been formally established for any of the genes in the region under consideration. Duplications could lead to overexpression of key genes in this region, disrupting normal neural development and function, possibly contributing to dystonia. Other duplications may involve additional genes that could lead to more pronounced neurodevelopmental delays and dysmorphic features, making more subtle or later-onset symptoms, such as dystonia, virtually undetectable.
(b) Alternatively, the association could involve a loss-of-function mechanism. For large duplications, this would require the breakpoint to disrupt one or more loci at the boundaries of the duplication. In this context, *MAGEE1* is an interesting candidate gene, as it is expressed in the CNS and located at the centromeric end of the duplication [17].

In the cases previously reported in the literature discussed above, all of the patients’ mothers carried the same microduplication, but three [12,13] did not show neurological abnormalities. Of these, only one [12] underwent X chromosome inactivation studies, which revealed 100% inactivation of the mutated chromosome. This underscores the importance of X inactivation mechanisms in shaping the phenotype in females.

By reporting a novel X chromosome duplication as the likely cause of a generalized dystonia phenotype, the authors highlight the critical role of identifying copy number variations and intragenic variants during genetic investigations. While WES has become a powerful tool in detecting single nucleotide variants and small indels, it may overlook larger genomic alterations, such as duplications or deletions, better identified through CMA. This way, the presented case emphasizes the necessity of integrating both approaches—WES and CMA—when evaluating patients with complex neurological phenotypes, including movement disorders, ensuring that larger structural variants (SVs) are not missed. Likely, the transition towards whole-genome sequencing will effectively enable the detection and characterization of SV breakpoints, including those that can be missed by WES and CMA (such as large inversions without loss of genetic information). This case report also serves as a reminder to consider skewed X chromosome inactivation as a potential mechanism underlying certain neurological phenotypes.

## Figures and Tables

**Table 1:** Summary of patient characteristics and corresponding X chromosome findings in the described family and published cases with duplications partially or totally overlapping the Xq13.3q21.1 region.

Patient	II7 (This Study)	III1 (This Study)	Martínez et al. [12], Patient 1	Martínez et al. [12], Patient 2	Chen et al. [13]	Sismani et al. [14]	Lugtenberg et al. [15]
**Sex**	Female	Male	Male	Male	Male	Male	Male
**Age of onset**	12	3	At birth	At birth	Gestation terminated (variant detected in fetus)		At birth
**Age at publication**	44	1	1	(Unknown)	-	-	26
**Movement disorder**	Hemidystonia, progressing to generalized dystonia		(Not described)				
**Other neurological features**	Mild intellectual delay; hyperreflexia; upper limb myoclonus	Learning disabilities; myoclonic epilepsy	Hypotonia; psychomotor delay; autistic traits	Hypotonia; psychomotor delay; ventriculomegaly; enlarged cortical sulci	-	Ventriculomegaly	Hypotonia; hypotrophy in infancy; feeding difficulties; psychomotor delay
**Dysmorphological and/or other features**	(No dysmorphological features) Cafe-au-lait spots; cerebral arteriovenous malformation rupture at 34 yo, with secondary epilepsy	(No dysmorphological features)		Postnatal growth deficiency; microcephaly; cryptorchidism; various craniofacial dysmorphisms	Hypoplastic male external genitals; clinodactyly; various craniofacial dysmorphisms	Nuchal translucency in the 1st trimester; polyhydramnios; increased weight and length; craniofacial and neck dysmorphisms; prominent thorax; single transverse palmar crease; hepatosplenomegaly	Short stature; craniofacial dysmorphisms; single palmar crease; clinodactyly; partial cutaneous syndactyly; genua valga; broad thorax and pectus excavatum; small penis and scrotum; cryptorchidism
**Array CGH result**	arr[hg19]Xq13.3q21.1(75640831-79709679)x3	(Not performed, the same duplication as in mother) [*]	arr[hg19]Xq13.3q21.1(65815490-78426724)x2	arr[hg19]Xq13.1q21.1(72433198-91125471)x2	arr[hg19]Xq13.3q21.1(75500269-76984168)x2	arr[hg19]Xq13.2q21.31(73187033–88124189)x2	arr[hg19]Xq13.2q21.1(72540999-79563235)x2 [**]
**Size of the duplication**	4 Mb		12.6 Mb	18.7 Mb	1.484 Mb	14.8 Mb	~7 Mb
**Family involvement of respective duplication**	Patient’s mother, sister, and four brothers are not carriers	Mother (II7)	Mother (no disease); maternal aunt not a carrier	Mother (no disease)	Mother and maternal grandmother (no disease)	Mother (skeletal abnormalities and altered speech)	Mother’s genetic testing was negative
**X Chromosome inactivation studies**	HUMARA assay identified skewed inactivation	(Not performed)	Mother: X inactivation pattern could not be determined	Mother: 100% inactivation of the X chromosome with the duplication	(Not described)		

Legend: array-CGH, Array-Comparative Genomic Hybridization; HUMARA, human androgen receptor gene; Mb, megabase; * Segregation studies were conducted using quantitative PCR (qPCR) with primers designed to anneal to genomic regions of the *PGK1* and *TBX22* genes. ** Approximate coordinates, mapped with BAC clones CTD-2003H23 and RP11-608A14.

## Data Availability

The data presented in this study are available on request from the corresponding author (data are not publicly available due to privacy restrictions).

## Supplementary Materials

The following supporting information can be downloaded at: https://www.mdpi.com/article/10.3390/ijms26020809/s1.

## References

[1] Figura M., Geremek M., Milanowski L.M., Meisner-Kramarz I., Duszynska-Was K., Szlufik S., Rozanski D., Smyk M., Koziorowski D. (2021). Movement disorders associated with chromosomal aberrations diagnosed in adult patients. Neurol. Neurochir. Pol..

[2] Dale R.C., Grattan-Smith P., Nicholson M., Peters G.B. (2012). Microdeletions detected using chromosome microarray in children with suspected genetic movement disorders: A single-centre study. Dev. Med. Child. Neurol..

[3] Garraux G., Caberg J.H., Vanbellinghen J.F., Jamar M., Bours V., Moonen G., Dive D. (2012). Partial trisomy 4q associated with young-onset dopa-responsive parkinsonism. Arch. Neurol..

[4] Carvalho V., Ferreira J.J., Correia Guedes L. (2021). Tremor and Parkinsonism in Chromosomopathies—A Systematic Review. Mov. Disord..

[5] Crosiers D., Blaumeiser B., Van Goethem G. (2019). Spectrum of Movement Disorders in 18p Deletion Syndrome. Mov. Disord. Clin. Pract..

[6] Keller Sarmiento I.J., Mencacci N.E. (2021). Genetic Dystonias: Update on Classification and New Genetic Discoveries. Curr. Neurol. Neurosci. Rep..

[7] Chin H.L., Lin C.Y., Chou O.H. (2023). X-linked dystonia parkinsonism: Epidemiology, genetics, clinical features, diagnosis, and treatment. Acta Neurol. Belg..

[8] Giacomini T., Vari M.S., Janis S., Prato G., Pisciotta L., Rocchi A., Michelucci A., Di Rocco M., Gandullia P., Mattioli G. (2019). Epileptic Encephalopathy, Myoclonus-Dystonia, and Premature Pubarche in Siblings with a Novel C-Terminal Truncating Mutation in ATRX Gene. Neuropediatrics.

[9] Harris J.C. (2018). Lesch-Nyhan syndrome and its variants: Examining the behavioral and neurocognitive phenotype. Curr. Opin. Psychiatry.

[10] Cordeiro D., Bullivant G., Siriwardena K., Evans A., Kobayashi J., Cohn R.D., Mercimek-Andrews S. (2018). Genetic landscape of pediatric movement disorders and management implications. Neurol. Genet..

[11] Allen R.C., Zoghbi H.Y., Moseley A.B., Rosenblatt H.M., Belmont J.W. (1992). Methylation of HpaII and HhaI sites near the polymorphic CAG repeat in the human androgen-receptor gene correlates with X chromosome inactivation. Am. J. Hum. Genet..

[12] Martinez F., Rosello M., Mayo S., Monfort S., Oltra S., Orellana C. (2014). Duplication at Xq13.3-q21.1 with syndromic intellectual disability, a probable role for the ATRX gene. Am. J. Med. Genet. A.

[13] Chen C.P., Yip H.K., Wang L.K., Chern S.R., Chen S.W., Lai S.T., Wu P.S., Wang W. (2017). Molecular genetic characterization of a prenatally detected 1.484-Mb Xq13.3-q21.1 duplication encompassing ATRX and a literature review of syndromic intellectual disability and congenital abnormalities in males with a duplication at Xq13.3-q21.1. Taiwan. J. Obstet. Gynecol..

[14] Sismani C., Donoghue J., Alexandrou A., Karkaletsi M., Christopoulou S., Konstantinidou A.E., Livanos P., Patsalis P.C., Velissariou V. (2013). A prenatally ascertained, maternally inherited 14.8 Mb duplication of chromosomal bands Xq13.2-q21.31 associated with multiple congenital abnormalities in a male fetus. Gene.

[15] Lugtenberg D., de Brouwer A.P., Oudakker A.R., Pfundt R., Hamel B.C., van Bokhoven H., Bongers E.M. (2009). Xq13.2q21.1 duplication encompassing the ATRX gene in a man with mental retardation, minor facial and genital anomalies, short stature and broad thorax. Am. J. Med. Genet. A.

[16] Kopanos C., Tsiolkas V., Kouris A., Chapple C.E., Albarca Aguilera M., Meyer R., Massouras A. (2019). VarSome: The human genomic variant search engine. Bioinformatics.

[17] Albrecht D.E., Froehner S.C. (2004). DAMAGE, a novel alpha-dystrobrevin-associated MAGE protein in dystrophin complexes. J. Biol. Chem..

