# Supplementary material for: A Novel Large Duplication on the X Chromosome as a Cause of Familial Generalized Dystonia: A Case Report
Source: Int J Mol Sci. 2025 Jan 19;26(2):809. doi: 10.3390/ijms26020809 (PMC11765987; doi:10.3390/ijms26020809)
Supplement: Supplementary file 1 [file ijms-26-00809-s001.zip › Supplementary data S1.pdf]

Supplementary Data S1

Next-generation sequencing methods

Whole-exome sequencing (WES) was performed using Agilent's SureSelect Human All Exon V6 capture kit and a HiSeq sequencer (Illumina). For data analysis, a custom-validated pipeline, based on the Broad Institute's Best Practices, was applied, using bwa-mem for alignment to the GRCh37 build of the human genome, GATK HaplotypeCaller for variant calling, and Ensembl VEP and GEMINI for variant annotation. Quality control (QC) was performed on the resulting FASTQ (FastQC), BAM (QualiMap and samtools) and VCF (bcftools) files, aggregated on a QC report using MultiQC. Variant calling was initially restricted to a virtual panel of 150 genes (Dystonia v7) and subsequently expanded to 4348 genes (Clinical exome v3) (please consider gene lists provided below). Copy Number Variants (CNVs) were called from WES data using a read depth-based algorithm supported by VarSeq software (Golden Helix, Inc). The read depth approach relies on detecting deviations in the expected read coverage across genomic regions, comparing with several dozens of WES samples, indicating potential large duplications or deletions. To ensure the accuracy and reliability of these calls, reportable CNVs identified by VarSeq are subsequently confirmed either by Multiplex Ligation-dependent Probe Amplification (MLPA) or, such as in this case, by quantitative Polymerase Chain Reaction (qPCR).

Variants were filtered by their minor allele frequency (MAF below 1% in population databases: NCBI's dbSNP, 1,000 Genome Project, Exome Variant Project, ExAC and gnomAD), for those that result in a change at the protein level and/or those previously described in the Human Gene Mutation Database (QIAGEN) or NCBI's ClinVar. All filtered single-nucleotide variants and small insertions-deletions were further analysed,

using Alamut Visual (Interactive Biosoftware) for in-silico pathogenicity predictions (including SIFT, PolyPhen2, CADD and splice-site prediction), population frequency confirmation and variants' visual inspection in the alignment file.

Virtual multigene panel – Dystonia v7:

AARS1, AARS2, ACTB, ADAR, ADCY5, AFG3L2, ALS2, ANO3, APTX, ARSA, ARX, ATM, ATP13A2, ATP1A2, ATP1A3, ATP7A, ATP7B, AUH, BCAP31, BCS1L, BSCL2, C19ORF12, CACNA1B, CIZ1, CLN3, COASY, COL4A1, COL6A3, COQ8A, COX10, COX15, COX20, CP, CSF1R, CYP27A1, DCAF17, DCTN1, DDC, DLAT, DNAJC12, DNAJC6, EARS2, ECHS1, FA2H, FBXO7, FOXG1, FOXRED1, FTL, FUCA1, GCDH, GCH1, GLB1, GM2A, GNAL, GNAO1, HACE1, HEPACAM, HEXA, HPCA, HPRT1, HTRA2, IFIH1, KCNMA1, KCTD17, KIF1C, KMT2B, LIPT1, MECP2, MECR, MICU1, MLC1, MMUT, MR1, NDUFA10, NDUFA12, NDUFA2, NDUFA9, NDUFAF2, NDUFAF5, NDUFAF6, NDUFS1, NDUFS2, NDUFS3, NDUFS4, NDUFS7, NDUFS8, NKX2-1, NPC1, NPC2, NUP62, PANK2, PDGFB, PDGFRB, PDHA1, PDHX, PINK1, PLA2G6, PLP1, PNKD, PNKP, PRKN, PRKRA, PRRT2, PSEN1, PTS, QDPR, RNASEH2A, RNASEH2B, RNASEH2C, SCN8A, SCP2, SDHA, SDHAF1, SERAC1, SGCE, SLC16A2, SLC19A3, SLC20A2, SLC25A15, SLC2A1, SLC30A10, SLC39A14, SLC6A3, SLC6A8, SMPD1, SPR, SQSTM1, STXBP1, SUCLA2, SUOX, SURF1, SYNJ1, TACO1, TAF1, TBC1D24, TH, THAP1, TIMM8A, TOR1A, TPI1, TPK1, TREX1, TTC19, TUBB4A, UBA5, UQCRQ, VAC14, VPS13A, WDR45, XPR1.

Virtual multigene panel – Clinical exome v3:

A2M A2ML1 AAAS AAGAB AARS1 AARS2 AASS ABAT ABCA1 ABCA12 ABCA3 ABCA4 ABCA5 ABCA7 ABCB1 ABCB11 ABCB4 ABCB6 ABCB7 ABCC2 ABCC6 ABCC8 ABCC9 ABCD1 ABCD3 ABCD4 ABCG5 ABCG8 ABHD12 ABHD5 ABL1 ACACB ACAD8 ACAD9 ACADL ACADM ACADS ACADSB ACADVL ACAN ACAT1 ACBD5 ACBD6 ACD ACE ACER3 ACO2 ACOX1 ACOX2 ACP4 ACP5 ACSF3 ACSL4 ACSL6 ACTA1 ACTA2 ACTB ACTC1 ACTG1 ACTG2 ACTL6A ACTL6B ACTN1 ACTN2 ACTN4 ACTRT1 ACVR1 ACVR1B ACVR2B ACVRL1 ACY1 ADA ADA2 ADAM10 ADAM17 ADAM22 ADAM9 ADAMTS10 ADAMTS13 ADAMTS17 ADAMTS18 ADAMTS2 ADAMTS3 ADAMTSL2 ADAMTSL4 ADAR ADAT3 ADCY1 ADCY10 ADCY3 ADCY5 ADCY6 ADD1 ADD3 ADGRA3 ADGRE2 ADGRG1 ADGRG2 ADGRG6 ADGRV1 ADH1C ADIPOQ ADIPOR1 ADK ADNP ADPRS ADRA2B ADRB2 ADRB3 ADSL ADSS1

AEBP1 AFF2 AFF3 AFF4 AFG3L2 AFP AGA AGBL1 AGBL5 AGK AGL AGO1 AGPAT2 AGPS AGRN AGRP AGT AGTR1 AGTR2 AGXT AHCY AHDC1 AHI1 AHR AHSG AICDA AIFM1 AIMP1 AIMP2 AIP AIPL1 AIRE AK1 AK2 AK7 AK9 AKAP10 AKAP9 AKR1C2 AKR1C4 AKR1D1 AKR1E2 AKT1 AKT2 AKT3 ALAD ALAS2 ALB ALDH18A1 ALDH1A2 ALDH1A3 ALDH2 ALDH3A2 ALDH4A1 ALDH5A1 ALDH6A1 ALDH7A1 ALDOA ALDOB ALG1 ALG10 ALG11 ALG12 ALG13 ALG14 ALG2 ALG3 ALG6 ALG8 ALG9 ALK ALMS1 ALOX12B ALOX5 ALOX5AP ALOXE3 ALPK3 ALPL ALS2 ALX1 ALX3 ALX4 AMACR AMBN AMELX AMER1 AMH AMHR2 AMMECR1 AMN AMOT AMPD1 AMPD2 AMT AMTN ANG ANGPTL3 ANGPTL4 ANGPTL6 ANK1 ANK2 ANK3 ANKFY1 ANKH ANKLE2 ANKRD1 ANKRD11 ANKRD17 ANKRD26 ANKS3 ANKS6 ANLN ANO10 ANO3 ANO5 ANO6 ANOS1 ANTXR1 ANTXR2 ANXA11 ANXA5 AP1S1 AP1S2 AP1S3 AP2S1 AP3B1 AP3B2 AP3D1 AP4B1 AP4E1 AP4M1 AP4S1 AP5Z1 APAF1 APC APC2 APCDD1 APOA1 APOA2 APOA5 APOB APOC2 APOC3 APOE APOL1 APP APPL1 APRT APTX AQP2 AQP4 AQP5 AR ARCN1 ARF1 ARFGEF2 ARG1 ARHGAP24 ARHGAP26 ARHGAP31 ARHGAP4 ARHGAP6 ARHGDIA ARHGEF10 ARHGEF15 ARHGEF18 ARHGEF2 ARHGEF28 ARHGEF6 ARHGEF9 ARID1A ARID1B ARID2 ARIH1 ARL13B ARL14EP ARL2BP ARL3 ARL6 ARL6IP1 ARL6IP6 ARMC4 ARMC5 ARMC9 ARMS2 ARNT2 ARPC1B ARR3 ARSA ARSB ARSG ARSI ARSL ARV1 ARX ASAH1 ASB10 ASCC1 ASCL1 ASH1L ASL ASMT ASNS ASPA ASPH ASPM ASPN ASPSCR1 ASS1 ASXL1 ASXL2 ASXL3 ATAD1 ATAD3A ATCAY ATF6 ATG16L1 ATG5 ATIC ATL1 ATL3 ATM ATN1 ATOH7 ATP11C ATP13A2 ATP1A1 ATP1A2 ATP1A3 ATP2A1 ATP2A2 ATP2B2 ATP2B3 ATP2B4 ATP2C1 ATP4A ATP5F1A ATP5F1D ATP5F1E ATP6AP1 ATP6AP2 ATP6V0A2 ATP6V0A4 ATP6V1A ATP6V1B1 ATP6V1B2 ATP6V1E1 ATP7A ATP7B ATP8A2 ATP8B1 ATPAF1 ATPAF2 ATR ATRIP ATRX ATXN1 ATXN10 ATXN2 ATXN3 ATXN7 AUH AURKA AURKC AUTS2 AVP AVPR2 AXIN1 AXIN2 B2M B3GALNT2 B3GALT6 B3GAT3 B3GLCT B4GALNT1 B4GALT1 B4GALT7 B4GAT1 B9D1 B9D2 BAAT BAG3 BANF1 BAP1 BARD1 BAX BBIP1 BBS1 BBS10 BBS12 BBS2 BBS4 BBS5 BBS7 BBS9 BCAP31 BCHE BCKDHA BCKDHB BCKDK BCL10 BCL11A BCL11B BCL2 BCL7A BCL9L BCO1 BCOR BCORL1 BCS1L BDNF BDP1 BEAN1 BEST1 BFSP1 BFSP2 BGN BHLHA9 BICC1 BICD2 BIN1 BLK BLM BLNK BLOC1S3 BLOC1S6 BLVRA BMP1 BMP15 BMP2 BMP4 BMP6 BMP7 BMPER BMPR1A BMPR1B BMPR2 BMS1 BOLA3 BPGM BPNT2 BPTF BRAF BRAT1 BRCA1 BRCA2 BRD2 BRD3 BRDT BRF1 BRIP1 BRPF1 BRWD3 BSCL2 BSN BSND BTD BTK BTNL2 BTRC BUB1 BUB1B BUB3 BVES C12orf4 C12orf57 C12orf65 C15orf41 C19orf12 C1GALT1C1 C1QA C1QB C1QBP C1QC C1QTNF5 C1R C1S C2 C2CD3 C3 C3AR1 C4A C4B C4BPA C4BPB C5 C5AR2 C6 C7 C8A C8B C8G

C8orf37 C9 C9orf72 CA12 CA2 CA4 CA5A CA5B CA8 CABP2 CABP4 CACNA1A CACNA1B CACNA1C CACNA1D CACNA1F CACNA1G CACNA1H CACNA1S CACNA2D1 CACNA2D2 CACNA2D4 CACNB2 CACNB4 CACNG2 CACNG3 CAD CALCR CALM1 CALM2 CALM3 CALR CALR3 CAMK2A CAMK2B CAMK2G CAMTA1 CANT1 CAPN1 CAPN10 CAPN3 CAPN5 CARD11 CARD14 CARD9 CARMIL2 CARS2 CARTPT CASK CASP10 CASP14 CASP2 CASP3 CASP8 CASQ1 CASQ2 CASR CAST CAT CATSPER1 CAV1 CAV3 CAVIN1 CBL CBLIF CBS CBX2 CC2D1A CC2D2A CCBE1 CCDC103 CCDC114 CCDC115 CCDC141 CCDC151 CCDC160 CCDC174 CCDC22 CCDC28B CCDC39 CCDC40 CCDC50 CCDC65 CCDC78 CCDC8 CCDC88A CCDC88C CCL2 CCM2 CCN6 CCND1 CCND2 CCNF CCNK CCNO CCNQ CCR5 CCT5 CD109 CD151 CD164 CD19 CD207 CD244 CD247 CD27 CD2AP CD320 CD36 CD3D CD3E CD3G CD4 CD40 CD40LG CD46 CD55 CD59 CD79A CD79B CD81 CD8A CD96 CD99L2 CDAN1 CDC14A CDC42 CDC42BPB CDC45 CDC6 CDC73 CDCA7 CDH1 CDH11 CDH15 CDH23 CDH3 CDHR1 CDK10 CDK13 CDK16 CDK4 CDK5 CDK5RAP2 CDK6 CDKL5 CDKN1A CDKN1B CDKN1C CDKN2A CDKN2B CDKN2C CDKN2D CDON CDSN CDT1 CEACAM16 CEBPA CEBPE CEL CELSR1 CELSR3 CENPE CENPF CENPJ CEP104 CEP120 CEP135 CEP152 CEP164 CEP19 CEP290 CEP41 CEP55 CEP57 CEP63 CEP78 CEP83 CEP89 CERKL CERS1 CERS3 CERT1 CES1 CETP CFAP298 CFAP300 CFAP410 CFAP43 CFAP44 CFAP52 CFAP53 CFAP69 CFB CFC1 CFD CFH CFHR1 CFHR3 CFHR4 CFHR5 CFI CFL2 CFP CFTR CHAMP1 CHAT CHCHD10 CHCHD2 CHD1 CHD2 CHD4 CHD7 CHD8 CHEK2 CHGB CHI3L1 CHIC2 CHKB CHL1 CHM CHMP1A CHMP2B CHMP4B CHN1 CHRDL1 CHRM3 CHRNA1 CHRNA2 CHRNA3 CHRNA4 CHRNA5 CHRNB1 CHRNB2 CHRND CHRNE CHRNG CHST11 CHST14 CHST3 CHST6 CHST8 CHSY1 CHUK CIB2 CIC CIDEC CIITA CILK1 CILP CISD2 CIT CITED2 CIZ1 CKAP2L CKM CLCF1 CLCN1 CLCN2 CLCN4 CLCN5 CLCN7 CLCNKA CLCNKB CLDN1 CLDN10 CLDN14 CLDN16 CLDN19 CLDN2 CLEC1A CLEC7A CLIC2 CLIC5 CLIP1 CLMP CLN3 CLN5 CLN6 CLN8 CLP1 CLPB CLPP CLPX CLRN1 CLTC CLTCL1 CLU CNBP CNGA1 CNGA2 CNGA3 CNGB1 CNGB3 CNKSR2 CNNM2 CNNM4 CNPY3 CNTN1 CNTN2 CNTNAP1 CNTNAP2 CNTNAP5 COA3 COA5 COA6 COA7 COA8 COASY COCH COG1 COG2 COG4 COG5 COG6 COG7 COG8 COL10A1 COL11A1 COL11A2 COL12A1 COL13A1 COL14A1 COL17A1 COL18A1 COL1A1 COL1A2 COL25A1 COL27A1 COL2A1 COL3A1 COL4A1 COL4A2 COL4A3 COL4A4 COL4A5 COL4A6 COL5A1 COL5A2 COL6A1 COL6A2 COL6A3 COL7A1 COL8A2 COL9A1 COL9A2 COL9A3 COLEC10 COLEC11 COLQ COMP COMT COPA COPB2 COQ2 COQ4 COQ5 COQ6 COQ7 COQ8A COQ8B COQ9 CORIN CORO1A COX10 COX14 COX15

COX20 COX4I1 COX4I2 COX5A COX6A1 COX6B1 COX7B COX8A CP CPA6 CPAMD8 CPLANE1 CPLX1 CPN1 CPOX CPS1 CPT1A CPT1C CPT2 CR2 CRADD CRAT CRB1 CRB2 CRBN CREB1 CREB3L1 CREB3L3 CREBBP CRELD1 CRH CRIM1 CRIPT CRLF1 CRPPA CRTAP CRTC1 CRX CRY1 CRYAA CRYAB CRYBA1 CRYBA2 CRYBA4 CRYBB1 CRYBB2 CRYBB3 CRYGB CRYGC CRYGD CRYGS CRYM CSF1R CSF2RA CSF2RB CSF3R CSNK1D CSNK1G1 CSNK2A1 CSNK2B CSPP1 CSRP3 CST3 CSTA CSTB CTBP1 CTC1 CTCF CTDP1 CTF1 CTH CTHRC1 CTLA4 CTNNA1 CTNNA2 CTNNA3 CTNNB1 CTNND1 CTNND2 CTNS CTPS1 CTRC CTSA CTSC CTSD CTSF CTSK CTTNBP2 CTU2 CUBN CUL3 CUL4B CUL7 CUX2 CWC27 CWF19L1 CX3CR1 CXCR2 CXCR4 CXorf56 CYB561 CYB5A CYB5R3 CYBA CYBB CYC1 CYCS CYFIP2 CYLD CYP11A1 CYP11B1 CYP11B2 CYP17A1 CYP19A1 CYP1B1 CYP21A2 CYP24A1 CYP26B1 CYP26C1 CYP27A1 CYP27B1 CYP2A6 CYP2C19 CYP2C9 CYP2D6 CYP2R1 CYP2U1 CYP3A5 CYP4F22 CYP4V2 CYP51A1 CYP7A1 CYP7B1 D2HGDH DAB1 DACH2 DACT1 DAG1 DAO DARS1 DARS2 DAXX DAZ2 DAZ3 DAZ4 DAZL DBH DBT DCAF17 DCAF8 DCC DCDC2 DCHS1 DCHS2 DCLRE1C DCN DCPS DCTN1 DCX DDAH1 DDB2 DDC DDHD1 DDHD2 DDOST DDR2 DDRGK1 DDX11 DDX3X DDX3Y DDX41 DDX58 DDX59 DEAF1 DENND5A DEPDC5 DES DGAT1 DGAT2 DGCR2 DGCR6 DGCR8 DGKE DGUOK DHCR24 DHCR7 DHDDS DHFR DHH DHODH DHTKD1 DHX30 DHX38 DIABLO DIAPH1 DIAPH2 DIAPH3 DICER1 DIP2B DIPK2B DIS3L2 DISC1 DISP1 DKC1 DKK1 DLAT DLC1 DLD DLG2 DLG3 DLG4 DLGAP2 DLL1 DLL3 DLL4 DLX3 DLX4 DLX5 DLX6 DMD DMGDH DMP1 DMPK DMRT1 DMRT3 DMXL2 DNA2 DNAAF1 DNAAF2 DNAAF3 DNAAF4 DNAAF5 DNAAF6 DNAH1 DNAH11 DNAH5 DNAH8 DNAI1 DNAI2 DNAJB11 DNAJB13 DNAJB2 DNAJB6 DNAJC12 DNAJC13 DNAJC19 DNAJC21 DNAJC3 DNAJC5 DNAJC6 DNAL1 DNAL4 DNASE1 DNASE1L3 DNM1 DNM1L DNM2 DNMT1 DNMT3A DNMT3B DOCK2 DOCK6 DOCK7 DOCK8 DOK7 DOLK DONSON DPAGT1 DPF2 DPH1 DPM1 DPM2 DPM3 DPP6 DPY19L2 DPYD DPYS DRAM2 DRC1 DRD2 DRD3 DRD4 DRD5 DRP2 DSC2 DSC3 DSE DSG1 DSG2 DSG4 DSP DSPP DST DSTYK DTHD1 DTNA DTNBP1 DUOX2 DUOXA2 DUSP6 DVL1 DVL2 DVL3 DYM DYNC1H1 DYNC2H1 DYNC2LI1 DYRK1A DYRK1B DYSF DZIP1L EARS2 EBF3 EBP ECE1 ECEL1 ECHS1 ECI1 ECM1 ECSIT EDA EDA2R EDAR EDARADD EDC3 EDN1 EDN3 EDNRA EDNRB EED EEF1A2 EEF1AKNMT EEF1B2 EEF2 EFEMP1 EFEMP2 EFHC1 EFHC2 EFL1 EFNA4 EFNB1 EFTUD2 EGF EGFR EGLN1 EGR1 EGR2 EHBP1 EHHADH EHMT1 EIF2AK3 EIF2AK4 EIF2B1 EIF2B2 EIF2B3 EIF2B4 EIF2B5 EIF2S3 EIF4A2 EIF4A3 EIF4E EIF4G1 ELAC2 ELANE ELF4 ELK1 ELMO2 ELMOD3 ELN ELOVL4 ELOVL5 ELP1 ELP2 ELP4 EMC1 EMD EMG1 EMILIN1 EML1 EMP2

EMX2 ENAM ENG ENO3 ENPP1 ENTPD1 EOGT EOMES EP300 EPAS1 EPB41 EPB41L1 EPB42 EPCAM EPG5 EPHA2 EPHA4 EPHB2 EPHB4 EPHX1 EPHX2 EPM2A EPO EPPK1 EPRS1 EPS15L1 EPS8 EPS8L2 EPS8L3 ERAL1 ERBB2 ERBB3 ERBB4 ERCC1 ERCC2 ERCC3 ERCC4 ERCC5 ERCC6 ERCC6L2 ERCC8 ERF ERGIC1 ERLIN1 ERLIN2 ERMARD ESCO2 ESPN ESR1 ESR2 ESRP1 ESRRB ETFA ETFB ETFDH ETHE1 ETV6 EVC EVC2 EWSR1 EXOSC2 EXOSC3 EXOSC8 EXOSC9 EXPH5 EXT1 EXT2 EXTL3 EYA1 EYA4 EYS EZH2 EZR F10 F11 F12 F13A1 F13B F2 F5 F7 F8 F9 FA2H FAAH2 FADD FAH FAM111A FAM111B FAM120C FAM126A FAM161A FAM20A FAM20C FAM83H FAN1 FANCA FANCB FANCC FANCD2 FANCE FANCF FANCG FANCI FANCL FANCM FAR1 FARS2 FARSB FAS FASLG FASN FASTKD2 FAT2 FAT4 FBLN5 FBN1 FBN2 FBP1 FBXL4 FBXO11 FBXO2 FBXO31 FBXO38 FBXO7 FBXW4 FCGR2A FCGR2B FCGR3A FCGR3B FCN3 FDFT1 FDPS FDX2 FDXR FECH FERMT1 FERMT3 FEZF1 FFAR4 FGA FGB FGD1 FGD4 FGF10 FGF12 FGF13 FGF14 FGF16 FGF17 FGF20 FGF23 FGF3 FGF5 FGF8 FGF9 FGFR1 FGFR2 FGFR3 FGG FH FHL1 FHL2 FIBP FIG4 FIGLA FKBP10 FKBP14 FKBP5 FKRP FKTN FLAD1 FLCN FLG FLG2 FLI1 FLII FLNA FLNB FLNC FLRT1 FLRT3 FLT3 FLT4 FLVCR1 FLVCR2 FMN1 FMN2 FMO3 FMR1 FN1 FNBP4 FOLR1 FOS FOXC1 FOXC2 FOXD3 FOXE1 FOXE3 FOXF1 FOXG1 FOXH1 FOXI1 FOXL2 FOXN1 FOXO1 FOXP1 FOXP2 FOXP3 FOXRED1 FPR1 FRAS1 FREM1 FREM2 FRG1 FRMD4A FRMD7 FRMPD4 FRRS1L FRY FRZB FSCN2 FSHB FSHR FSIP2 FTCD FTH1 FTL FTO FTSJ1 FUCA1 FUS FUT6 FUT8 FUZ FXN FXYD2 FYB1 FYCO1 FZD2 FZD3 FZD4 FZD5 FZD6 G6PC G6PC3 G6PD GAA GAB1 GABBR2 GABRA1 GABRB1 GABRB2 GABRB3 GABRD GABRE GABRG1 GABRG2 GAD1 GAL GALC GALE GALK1 GALNS GALNT12 GALNT3 GALT GAMT GAN GANAB GAPVD1 GARS1 GAS1 GAS8 GATA1 GATA2 GATA3 GATA4 GATA5 GATA6 GATAD1 GATAD2B GATM GBA GBA2 GBE1 GCDH GCGR GCH1 GCK GCLC GCLM GCM2 GCNT2 GCSH GDAP1 GDF1 GDF2 GDF3 GDF5 GDF6 GDF9 GDI1 GDNF GEMIN4 GFAP GFER GFI1 GFI1B GFM1 GFPT1 GGCX GGT1 GH1 GHR GHRHR GHRL GHSR GIGYF2 GINS1 GIPC3 GJA1 GJA3 GJA5 GJA8 GJB1 GJB2 GJB3 GJB4 GJB6 GJC2 GK GLA GLB1 GLDC GLDN GLE1 GLI1 GLI2 GLI3 GLIS2 GLIS3 GLMN GLRA1 GLRA2 GLRB GLRX5 GLUD1 GLUD2 GLUL GLYCTK GM2A GMNN GMPPA GMPPB GNA11 GNAI1 GNAI2 GNAI3 GNAL GNAO1 GNAQ GNAS GNAT1 GNAT2 GNB1 GNB3 GNB4 GNB5 GNE GNMT GNPAT GNPTAB GNPTG GNRH1 GNRHR GNS GON4L GOPC GORAB GOSR2 GOT1 GP1BA GP1BB GP6 GP9 GPAA1 GPC3 GPC4 GPC6 GPD1 GPD1L GPD2 GPHN GPI GPIHBP1 GPKOW GPNMB GPR101 GPR143 GPR161 GPR179 GPR180 GPR68 GPR88 GPRASP2 GPSM2 GPT2 GPX4 GREB1L GREM1 GREM2

GRHL2 GRHL3 GRHPR GRIA1 GRIA2 GRIA3 GRIA4 GRID2 GRIK2 GRIN1 GRIN2A GRIN2B GRIN2D GRIP1 GRIPAP1 GRK1 GRM1 GRM6 GRN GRPR GRXCR1 GRXCR2 GSC GSDME GSN GSPT2 GSS GTF2E2 GTF2H5 GTPBP2 GTPBP3 GUCA1A GUCA1B GUCY1A1 GUCY2C GUCY2D GUF1 GUSB GYG1 GYG2 GYS1 GYS2 GZF1 H1-4 H2BW2 H3-4 H6PD HAAO HABP2 HACD1 HACE1 HADH HADHA HADHB HAMP HAND1 HARS1 HARS2 HAX1 HBA1 HBA2 HBB HBD HBG1 HBG2 HCCS HCFC1 HCN1 HCN2 HCN4 HCRT HDAC4 HDAC6 HDAC8 HDC HECW2 HELLS HEPACAM HEPH HERC1 HERC2 HES7 HESX1 HEXA HEXB HFE HFM1 HGD HGF HGSNAT HHAT HIBCH HIC1 HIKESHI HINT1 HIVEP2 HJV HK1 HK2 HLCS HMBS HMCN1 HMGA1 HMGB3 HMGCL HMGCS2 HMMR HMOX1 HMX1 HNF1A HNF1B HNF4A HNMT HNRNPA1 HNRNPA2B1 HNRNPDL HNRNPH2 HNRNPK HNRNPU HOGA1 HOMER2 HOXA1 HOXA11 HOXA13 HOXA2 HOXB1 HOXB13 HOXC13 HOXD10 HOXD13 HPCA HPD HPGD HPRT1 HPS1 HPS3 HPS4 HPS5 HPS6 HPSE2 HR HRAS HRG HS6ST1 HSD11B1 HSD11B2 HSD17B10 HSD17B3 HSD17B4 HSD3B2 HSD3B7 HSF4 HSPA9 HSPB1 HSPB3 HSPB8 HSPD1 HSPG2 HTR1A HTRA1 HTRA2 HTT HUWE1 HYAL1 HYAL2 HYDIN HYLS1 IARS1 IARS2 IBA57 ICOS IDH1 IDH2 IDH3B IDS IDUA IER3IP1 IFIH1 IFITM5 IFNAR2 IFNG IFNGR1 IFNGR2 IFRD1 IFT122 IFT140 IFT172 IFT27 IFT43 IFT52 IFT57 IFT74 IFT80 IFT81 IFT88 IGBP1 IGF1 IGF1R IGF2 IGF2BP2 IGF2R IGFALS IGFBP7 IGHMBP2 IGLL1 IGSF1 IGSF3 IHH IKBKB IKBKG IKZF1 IL10 IL10RA IL10RB IL11RA IL12B IL12RB1 IL13 IL17F IL17RA IL17RC IL17RD IL1RAPL1 IL1RN IL21 IL21R IL23R IL2RA IL2RG IL31RA IL36RN IL4R IL6 IL7R ILDR1 ILK IMMP2L IMPA1 IMPDH1 IMPG1 IMPG2 INAVA INF2 ING1 INHBA INPP4A INPP5E INPP5K INPPL1 INS INSL3 INSR INTU INVS IPMK IQCB1 IQCE IQSEC2 IRAK4 IRF1 IRF2BP2 IRF2BPL IRF3 IRF5 IRF6 IRF7 IRF8 IRGM IRS1 IRS2 IRX4 IRX5 ISCA1 ISCA2 ISCU ISG15 ITCH ITGA2B ITGA3 ITGA6 ITGA7 ITGA8 ITGAM ITGB2 ITGB3 ITGB4 ITGB6 ITIH4 ITK ITM2B ITPA ITPKC ITPR1 ITPR2 IVD IYD JAG1 JAG2 JAGN1 JAK2 JAK3 JAM3 JPH1 JPH2 JPH3 JRK JUP KALRN KANK1 KANK2 KANSL1 KARS1 KAT6A KAT6B KATNAL2 KATNB1 KBTBD13 KCNA1 KCNA2 KCNA5 KCNB1 KCNC1 KCNC3 KCND3 KCNE1 KCNE2 KCNE3 KCNE5 KCNH1 KCNH2 KCNH5 KCNJ1 KCNJ10 KCNJ11 KCNJ13 KCNJ18 KCNJ2 KCNJ5 KCNJ6 KCNJ8 KCNK18 KCNK3 KCNK9 KCNMA1 KCNMB1 KCNN4 KCNQ1 KCNQ2 KCNQ3 KCNQ4 KCNQ5 KCNT1 KCNT2 KCNV2 KCTD1 KCTD13 KCTD17 KCTD7 KDF1 KDM1A KDM5A KDM5B KDM5C KDM6A KDM6B KDR KDSR KEAP1 KERA KHDC3L KIAA0556 KIAA0586 KIAA0753 KIAA1109 KIDINS220 KIF11 KIF14 KIF15 KIF1A KIF1B KIF1C KIF20A KIF21A KIF22 KIF23 KIF26B KIF2A KIF4A KIF5A KIF5C KIF7 KIFBP KIRREL3 KISS1 KISS1R KIT KITLG KIZ KL

KLC2 KLC4 KLF1 KLF11 KLF6 KLF8 KLHDC8B KLHL10 KLHL13 KLHL15 KLHL24 KLHL3 KLHL40 KLHL41 KLHL7 KLHL9 KLK4 KLKB1 KLLN KMT2A KMT2B KMT2C KMT2D KMT5B KNL1 KNSTRN KPNA7 KPTN KRAS KREMEN1 KRIT1 KRT1 KRT10 KRT12 KRT13 KRT14 KRT16 KRT17 KRT2 KRT25 KRT3 KRT4 KRT5 KRT6A KRT6B KRT6C KRT71 KRT74 KRT75 KRT81 KRT83 KRT85 KRT86 KRT9 KY KYNU L1CAM L2HGDH LACC1 LAGE3 LAMA1 LAMA2 LAMA3 LAMA4 LAMB1 LAMB2 LAMB3 LAMC2 LAMC3 LAMP2 LAMTOR2 LARGE1 LARP7 LARS1 LARS2 LAS1L LAT LBR LCA5 LCAT LCK LCT LDB3 LDHA LDHB LDLR LDLRAP1 LEF1 LEMD2 LEMD3 LEP LEPR LFNG LGALS2 LGI1 LGI4 LHB LHCGR LHFPL5 LHX3 LHX4 LIAS LIFR LIG4 LIM2 LIMS2 LINGO1 LINS1 LIPA LIPC LIPE LIPH LIPN LIPT1 LIPT2 LITAF LMAN1 LMAN2L LMBR1 LMBRD1 LMF1 LMNA LMNB1 LMNB2 LMOD1 LMOD3 LMX1A LMX1B LNPK LONP1 LORICRIN LOX LOXHD1 LOXL1 LOXL3 LPA LPAR6 LPIN1 LPIN2 LPL LPP LRAT LRBA LRIG2 LRIT3 LRMDA LRP1 LRP2 LRP4 LRP5 LRP6 LRP8 LRPAP1 LRPPRC LRRC40 LRRC6 LRRC8A LRRK1 LRRK2 LRSAM1 LRTOMT LSS LTA LTBP2 LTBP3 LTBP4 LUM LYRM4 LYRM7 LYST LYZ LZTFL1 LZTR1 LZTS1 MAB21L2 MAD1L1 MAD2L2 MAF MAFA MAFB MAG MAGEA11 MAGEA4 MAGED2 MAGEL2 MAGI2 MAGT1 MAK MALT1 MAML2 MAMLD1 MAN1B1 MAN2B1 MANBA MAOA MAP1B MAP2K1 MAP2K2 MAP3K1 MAP3K14 MAP3K15 MAP3K20 MAP3K6 MAP3K7 MAP3K8 MAP7D3 MAPK10 MAPK8IP1 MAPKAPK3 MAPKBP1 MAPRE2 MAPT MARS1 MARS2 MARVELD2 MASP1 MASP2 MASTL MAT1A MAT2A MATN3 MATR3 MAX MBD5 MBNL3 MBOAT7 MBTPS2 MC1R MC2R MC4R MCC MCCC1 MCCC2 MCEE MCFD2 MCIDAS MCM2 MCM3AP MCM4 MCM5 MCM6 MCM8 MCM9 MCOLN1 MCPH1 MDH2 MDM2 ME2 MECOM MECP2 MECR MED12 MED13L MED17 MED23 MED25 MEF2A MEF2C MEFV MEGF10 MEGF8 MEIOB MEIS2 MEN1 MEOX1 MERTK MESP2 MET METTL23 MFAP5 MFF MFN2 MFRP MFSD2A MFSD8 MGAT2 MGME1 MGP MIB1 MICOS13 MICU1 MID1 MID2 MIF MINPP1 MIP MIPEP MITF MKKS MKRN3 MKS1 MLC1 MLH1 MLH3 MLLT10 MLPH MLXIPL MLYCD MMAA MMAB MMACHC MMADHC MME MMP1 MMP13 MMP14 MMP19 MMP2 MMP20 MMP21 MMP3 MMP9 MMUT MN1 MNX1 MOCOS MOCS1 MOCS2 MOG MOGS MORC2 MPC1 MPDU1 MPDZ MPI MPIG6B MPL MPLKIP MPO MPV17 MPZ MPZL2 MRAP MRAP2 MRE11 MRPL3 MRPL44 MRPS16 MRPS2 MRPS22 MRPS34 MRPS7 MRTFA MS4A1 MS4A2 MSH2 MSH3 MSH5 MSH6 MSMB MSMO1 MSN MSR1 MSRB3 MST1 MST1R MSTN MSTO1 MSX1 MSX2 MTAP MTF1 MTFMT MTHFD1 MTHFR MTM1 MTMR14 MTMR2 MTNR1B MTO1 MTOR MTPAP MTR MTRR MTTP MUC1 MUC5B MUSK MUTYH MVD MVK MXI1 MYB MYBPC1 MYBPC3 MYC MYCN MYD88 MYF5 MYF6 MYH11 MYH14 MYH2 MYH3 MYH6

MYH7 MYH7B MYH8 MYH9 MYL2 MYL3 MYL4 MYLK MYLK2 MYMK MYO15A MYO18B MYO1A MYO1C MYO1E MYO1F MYO1G MYO1H MYO3A MYO5A MYO5B MYO6 MYO7A MYO9A MYO9B MYOC MYOD1 MYOM1 MYOT MYOZ2 MYPN MYRF MYSM1 MYT1L NAA10 NAA15 NACC1 NADK2 NAGA NAGLU NAGS NALCN NANOS1 NANS NAP1L3 NARS2 NAT10 NAT8L NAXE NBAS NBEAL2 NBN NCAPD2 NCAPD3 NCAPH NCF1 NCF2 NCF4 NCSTN NDE1 NDN NDP NDRG1 NDST1 NDUFA1 NDUFA10 NDUFA11 NDUFA12 NDUFA13 NDUFA2 NDUFA4 NDUFA6 NDUFA7 NDUFA8 NDUFA9 NDUFAF1 NDUFAF2 NDUFAF3 NDUFAF4 NDUFAF5 NDUFAF6 NDUFB1 NDUFB10 NDUFB11 NDUFB3 NDUFB8 NDUFB9 NDUFS1 NDUFS2 NDUFS3 NDUFS4 NDUFS5 NDUFS6 NDUFS7 NDUFS8 NDUFV1 NDUFV2 NDUFV3 NEB NEBL NECAP1 NECTIN1 NECTIN4 NEDD4L NEFH NEFL NEGR1 NEK1 NEK2 NEK8 NEK9 NELFA NEU1 NEUROD1 NEUROG1 NEUROG3 NEXMIF NEXN NF1 NF2 NFE2L2 NFIA NFIX NFKB1 NFKB2 NFKBIA NFKBIL1 NFS1 NFU1 NGF NGLY1 NHEJ1 NHLRC1 NHP2 NHS NIN NIPA1 NIPA2 NIPAL4 NIPBL NIPSNAP1 NIPSNAP3A NKX2-1 NKX2-5 NKX2-6 NKX3-2 NKX6-2 NLGN3 NLGN4X NLRC4 NLRP1 NLRP12 NLRP3 NLRP7 NME1 NME7 NME8 NMNAT1 NNT NOBOX NOD2 NODAL NOG NOL3 NONO NOP10 NOP56 NOS1 NOS2 NOS3 NOTCH1 NOTCH2 NOTCH3 NPC1 NPC2 NPHP1 NPHP3 NPHP4 NPHS1 NPHS2 NPL NPM1 NPPA NPPC NPR2 NPRL2 NPRL3 NR0B1 NR0B2 NR1H4 NR1I3 NR2E3 NR2F1 NR2F2 NR3C1 NR3C2 NR4A2 NR4A3 NR5A1 NRAS NRL NRXN1 NRXN2 NRXN3 NSD1 NSDHL NSMCE2 NSMCE3 NSMF NSUN2 NT5C2 NT5C3A NT5E NTF4 NTHL1 NTM NTN1 NTNG1 NTRK1 NTRK2 NUBPL NUMA1 NUP107 NUP133 NUP155 NUP160 NUP205 NUP214 NUP37 NUP62 NUP85 NUP93 NUS1 NXF5 NXN NYX OAS1 OAT OBSCN OBSL1 OCA2 OCLN OCRL ODAPH ODF2L OFD1 OGG1 OGT OLR1 OPA1 OPA3 OPHN1 OPLAH OPN1LW OPN1MW OPN1SW OPTN OR5M1 ORAI1 ORC1 ORC4 ORC6 OSBPL2 OSGEP OSMR OSTM1 OTC OTOA OTOF OTOG OTOGL OTOR OTUD6B OTULIN OTX2 OVOL2 OXCT1 P2RX2 P2RY12 P3H1 P3H2 P4HA1 P4HA2 P4HB PABPN1 PACRG PACS1 PACS2 PADI3 PADI4 PADI6 PAFAH1B1 PAH PAK1 PAK3 PAK5 PALB2 PALLD PAM16 PANK2 PAPSS2 PARK7 PARL PARN PARP1 PATL2 PAX1 PAX2 PAX3 PAX4 PAX5 PAX6 PAX7 PAX8 PAX9 PBX1 PC PCARE PCBD1 PCCA PCCB PCDH10 PCDH11X PCDH12 PCDH15 PCDH19 PCDH9 PCGF2 PCK1 PCLO PCNA PCNT PCSK1 PCSK9 PCYT1A PDCD1 PDCD10 PDE10A PDE11A PDE1C PDE3A PDE4D PDE6A PDE6B PDE6C PDE6D PDE6G PDE6H PDE8B PDGFB PDGFRA PDGFRB PDGFRL PDHA1 PDHB PDHX PDK3 PDLIM3 PDLIM4 PDP1 PDSS1 PDSS2 PDX1 PDYN PDZD7 PECR PEPD PER2 PER3 PET100 PET117 PEX1 PEX10 PEX11B PEX12 PEX13 PEX14 PEX16 PEX19 PEX2 PEX26 PEX3 PEX5 PEX6 PEX7 PFKM

PFN1 PGAM2 PGAP1 PGAP2 PGAP3 PGK1 PGM1 PGM3 PHACTR1 PHB PHC1 PHEX PHF10 PHF6 PHF8 PHGDH PHIP PHKA1 PHKA2 PHKB PHKG1 PHKG2 PHOX2A PHOX2B PHYH PI4KA PIBF1 PICALM PICK1 PIEZO1 PIEZO2 PIGA PIGC PIGG PIGH PIGL PIGM PIGN PIGO PIGP PIGQ PIGS PIGT PIGV PIGW PIGY PIK3AP1 PIK3CA PIK3CD PIK3R1 PIK3R2 PIK3R5 PIKFYVE PIN1 PINK1 PIP5K1C PITPNM3 PITX1 PITX2 PITX3 PJVK PKD1 PKD1L1 PKD2 PKHD1 PKLR PKP1 PKP2 PLA2G2A PLA2G4A PLA2G6 PLA2G7 PLAA PLAG1 PLAU PLCB1 PLCB4 PLCD1 PLCE1 PLCG2 PLCZ1 PLD1 PLD3 PLEC PLEKHG2 PLEKHG4 PLEKHG5 PLEKHM1 PLG PLIN1 PLK4 PLN PLOD1 PLOD2 PLOD3 PLP1 PLP2 PLPBP PLPP6 PLS3 PLVAP PLXNA3 PLXNB3 PLXND1 PMFBP1 PML PMM2 PMP2 PMP22 PMPCA PMPCB PMS1 PMS2 PMVK PNKD PNKP PNLIP PNP PNPLA1 PNPLA2 PNPLA4 PNPLA6 PNPLA8 PNPO PNPT1 POC1A POC1B PODXL POF1B POFUT1 POGLUT1 POGZ POLA1 POLD1 POLE POLG POLG2 POLH POLR1A POLR1C POLR1D POLR3A POLR3B POMC POMGNT1 POMGNT2 POMK POMP POMT1 POMT2 PON1 PON2 PON3 POP1 POR PORCN POT1 POU1F1 POU3F4 POU4F3 POU6F2 PPA2 PPARA PPARG PPARGC1B PPCS PPIB PPL PPM1D PPM1K PPOX PPP1CB PPP1R15B PPP1R17 PPP1R3A PPP2R1A PPP2R1B PPP2R2B PPP2R5D PPP3CA PPT1 PQBP1 PRCC PRCD PRDM12 PRDM16 PRDM5 PRDM6 PRDM8 PRDX1 PREPL PRF1 PRG4 PRICKLE1 PRICKLE2 PRICKLE3 PRIMPOL PRKACA PRKACG PRKAG2 PRKAR1A PRKAR1B PRKCA PRKCD PRKCG PRKCH PRKCSH PRKD1 PRKDC PRKG1 PRKN PRKRA PRLR PRMT7 PRNP PROC PRODH PROK2 PROKR2 PROM1 PROP1 PROS1 PROX2 PROZ PRPF3 PRPF31 PRPF4 PRPF6 PRPF8 PRPH PRPH2 PRPS1 PRRT2 PRRX1 PRSS12 PRSS56 PRUNE1 PRX PSAP PSAT1 PSEN1 PSEN2 PSENEN PSMA6 PSMA7 PSMB4 PSMB8 PSMB9 PSMC3IP PSMD12 PSPH PSTPIP1 PTCH1 PTCH2 PTCHD1 PTDSS1 PTEN PTF1A PTGIS PTH PTH1R PTHLH PTK7 PTPN1 PTPN11 PTPN12 PTPN14 PTPN2 PTPN22 PTPRC PTPRF PTPRJ PTPRO PTPRQ PTRH2 PTS PUF60 PUM1 PURA PUS1 PUS3 PXDN PYCR1 PYCR2 PYGL PYGM PYROXD1 QARS1 QDPR QRICH1 RAB11A RAB11B RAB18 RAB23 RAB27A RAB28 RAB29 RAB33B RAB39B RAB3GAP1 RAB3GAP2 RAB40AL RAB7A RABL6 RAC1 RAC2 RAD21 RAD50 RAD51 RAD51C RAD51D RAD54B RAD54L RAF1 RAG1 RAG2 RAI1 RALGDS RANBP2 RANGAP1 RANGRF RAP1A RAP1B RAP1GDS1 RAPGEF1 RAPGEF2 RAPSN RARB RARS1 RARS2 RASA1 RASA2 RASGRP1 RASGRP2 RAX RAX2 RB1 RB1CC1 RBBP8 RBCK1 RBFOX1 RBFOX3 RBM10 RBM12 RBM20 RBM28 RBM4B RBM8A RBMX RBMY1A1 RBP3 RBP4 RBPJ RBSN RCBTB1 RCN2 RD3 RDH11 RDH12 RDH5 RDX RECQL4 REEP1 REEP2 REEP6 RELB RELN REN REPS1 RERE REST RET RETN RETREG1 REV3L RFT1 RFWD3 RFX5 RFX6 RFXANK RFXAP RGR

RGS6 RGS7 RGS9 RGS9BP RHAG RHBDF2 RHEB RHO RHOBTB2 RHOH RIMS1 RIN2 RIPK1 RIPK2 RIPK4 RIPOR2 RIPPLY1 RIPPLY2 RIT1 RLBP1 RLIM RMI2 RMND1 RNASEH1 RNASEH2A RNASEH2B RNASEH2C RNASEL RNASET2 RNF113A RNF125 RNF128 RNF135 RNF139 RNF168 RNF170 RNF212 RNF213 RNF216 RNF31 RNF43 RNF6 RNLS RNPC3 ROBO1 ROBO2 ROBO3 ROGDI ROM1 ROR1 ROR2 RORA RORC RP1 RP1L1 RP2 RP9 RPE65 RPGR RPGRIP1 RPGRIP1L RPIA RPL10 RPL11 RPL15 RPL18 RPL21 RPL26 RPL27 RPL35 RPL35A RPL5 RPS10 RPS14 RPS17 RPS19 RPS20 RPS23 RPS24 RPS26 RPS27 RPS28 RPS29 RPS6KA3 RPS7 RPSA RRAS RRAS2 RRM2B RS1 RSPH1 RSPH3 RSPH4A RSPH9 RSPO1 RSPO2 RSPO4 RSPRY1 RTEL1 RTN2 RTN4IP1 RTN4R RTTN RUBCN RUNX1 RUNX2 RUSC2 RXYLT1 RYR1 RYR2 RYR3 S1PR2 SACS SAG SALL1 SALL2 SALL4 SAMD12 SAMD9 SAMD9L SAMHD1 SAR1B SARS1 SARS2 SASH1 SASS6 SAT1 SATB2 SBDS SBF1 SBF2 SC5D SCAPER SCARB2 SCARF2 SCLT1 SCN10A SCN11A SCN1A SCN1B SCN2A SCN2B SCN3A SCN3B SCN4A SCN4B SCN5A SCN8A SCN9A SCNN1A SCNN1B SCNN1G SCO1 SCO2 SCP2 SCRIB SCYL1 SDC3 SDCCAG8 SDHA SDHAF1 SDHAF2 SDHB SDHC SDHD SDR9C7 SEC23A SEC23B SEC24D SEC61A1 SEC63 SECISBP2 SELENBP1 SELENOI SELENON SEM1 SEMA3A SEMA3E SEMA4A SEMA5A SEPSECS SEPTIN12 SEPTIN6 SEPTIN9 SERAC1 SERPINA1 SERPINA3 SERPINA6 SERPINB6 SERPINB7 SERPINB8 SERPINC1 SERPIND1 SERPINE1 SERPINF1 SERPINF2 SERPING1 SERPINH1 SERPINI1 SET SETBP1 SETD1A SETD2 SETD5 SETX SF3B1 SF3B4 SFRP4 SFTPA2 SFTPB SFTPC SFXN4 SGCA SGCB SGCD SGCE SGCG SGO1 SGPL1 SGSH SH2B1 SH2B3 SH2D1A SH3BP2 SH3KBP1 SH3PXD2B SH3TC2 SHANK1 SHANK2 SHANK3 SHH SHOC2 SHOX SHROOM2 SHROOM3 SHROOM4 SI SIAE SIGMAR1 SIK1 SIK3 SIL1 SIM1 SIN3A SIPA1L3 SIRT3 SIX1 SIX3 SIX5 SIX6 SKI SKIV2L SLC10A2 SLC11A2 SLC12A1 SLC12A2 SLC12A3 SLC12A5 SLC12A6 SLC13A5 SLC16A1 SLC16A12 SLC16A2 SLC17A5 SLC17A8 SLC17A9 SLC18A2 SLC18A3 SLC19A2 SLC19A3 SLC1A1 SLC1A2 SLC1A3 SLC1A4 SLC20A2 SLC22A12 SLC22A18 SLC22A4 SLC22A5 SLC24A1 SLC24A4 SLC24A5 SLC25A1 SLC25A12 SLC25A13 SLC25A15 SLC25A19 SLC25A20 SLC25A22 SLC25A24 SLC25A26 SLC25A3 SLC25A32 SLC25A38 SLC25A4 SLC25A46 SLC25A5 SLC26A1 SLC26A2 SLC26A3 SLC26A4 SLC26A5 SLC26A8 SLC26A9 SLC27A4 SLC27A5 SLC29A3 SLC2A1 SLC2A10 SLC2A2 SLC2A9 SLC30A10 SLC30A2 SLC30A8 SLC30A9 SLC31A1 SLC33A1 SLC34A1 SLC34A2 SLC34A3 SLC35A1 SLC35A2 SLC35A3 SLC35C1 SLC35D1 SLC36A2 SLC37A4 SLC38A8 SLC39A13 SLC39A14 SLC39A4 SLC39A5 SLC39A8 SLC3A1 SLC40A1 SLC44A4 SLC45A1 SLC45A2 SLC46A1 SLC49A4 SLC4A1 SLC4A10 SLC4A11 SLC4A4 SLC52A1 SLC52A2 SLC52A3 SLC5A1 SLC5A2 SLC5A5 SLC5A7 SLC6A1 SLC6A14 SLC6A17

SLC6A18 SLC6A19 SLC6A2 SLC6A20 SLC6A3 SLC6A4 SLC6A5 SLC6A8 SLC6A9 SLC7A14 SLC7A3 SLC7A7 SLC7A9 SLC9A1 SLC9A3 SLC9A3R1 SLC9A6 SLC9A9 SLCO1B1 SLCO1B3 SLCO2A1 SLFN14 SLITRK1 SLITRK6 SLMAP SLURP1 SLX4 SMAD2 SMAD3 SMAD4 SMAD6 SMAD7 SMAD9 SMARCA1 SMARCA2 SMARCA4 SMARCAD1 SMARCAL1 SMARCB1 SMARCC1 SMARCC2 SMARCD1 SMARCD2 SMARCE1 SMC1A SMC3 SMCHD1 SMG9 SMN1 SMN2 SMO SMOC1 SMOC2 SMPD1 SMPX SMS SNAI2 SNAP25 SNAP29 SNCA SNCAIP SNCB SNIP1 SNRNP200 SNRPB SNRPE SNRPN SNTA1 SNX10 SNX14 SOBP SOD1 SOD2 SOHLH1 SON SORL1 SOS1 SOS2 SOST SOX10 SOX11 SOX17 SOX18 SOX2 SOX3 SOX5 SOX6 SOX9 SP110 SP7 SPAG1 SPAG17 SPANXC SPARC SPART SPAST SPATA16 SPATA5 SPATA7 SPECC1L SPEG SPG11 SPG21 SPG7 SPIDR SPIN3 SPINK1 SPINK2 SPINK5 SPINT2 SPP2 SPR SPRED1 SPRTN SPRY2 SPRY4 SPTA1 SPTAN1 SPTB SPTBN2 SPTBN4 SPTLC1 SPTLC2 SQSTM1 SRC SRCAP SRD5A2 SRD5A3 SRGAP1 SRGAP2 SRP54 SRP72 SRPK3 SRPX SRPX2 SRY SSR4 SSTR5 SSUH2 SSX1 SSX2 ST14 ST3GAL3 ST3GAL5 ST7 STAB2 STAC3 STAG1 STAG2 STAG3 STAMBP STAR STAT1 STAT2 STAT3 STAT4 STAT5B STEAP3 STIL STIM1 STING1 STK11 STK3 STK36 STK4 STN1 STOX1 STRA6 STRADA STRC STS STT3A STT3B STUB1 STX11 STX16 STX1B STX3 STXBP1 STXBP2 SUCLA2 SUCLG1 SUCLG2 SUFU SUGCT SULF1 SULT2B1 SUMF1 SUMO1 SUMO4 SUN5 SUOX SURF1 SUZ12 SYCE1 SYCP3 SYN1 SYN2 SYNCRIP SYNE1 SYNE2 SYNE4 SYNGAP1 SYNJ1 SYNJ2 SYP SYT1 SYT14 SYT2 SYTL4 SZT2 TAB2 TAC3 TACO1 TACR3 TACSTD2 TAF1 TAF13 TAF1A TAF2 TAF4B TAF6 TAL1 TAL2 TALDO1 TANC2 TANGO2 TAP1 TAP2 TAPBP TAPT1 TARDBP TARS2 TAT TAZ TBC1D20 TBC1D23 TBC1D24 TBC1D32 TBC1D4 TBC1D7 TBCD TBCE TBCK TBK1 TBL1X TBL1XR1 TBL2 TBP TBR1 TBX1 TBX10 TBX15 TBX18 TBX19 TBX20 TBX21 TBX22 TBX3 TBX4 TBX5 TBX6 TBXA2R TBXAS1 TBXT TCAP TCF12 TCF20 TCF21 TCF3 TCF4 TCF7L2 TCHH TCIRG1 TCN2 TCOF1 TCTEX1D2 TCTN1 TCTN2 TCTN3 TDGF1 TDP1 TDP2 TDRD7 TDRD9 TEAD1 TECPR2 TECR TECRL TECTA TEK TELO2 TENM1 TENM3 TENM4 TENT5A TERT TET1 TET2 TEX11 TEX14 TEX15 TF TFAM TFAP2A TFAP2B TFE3 TFG TFR2 TFRC TG TGDS TGFA TGFB1 TGFB2 TGFB3 TGFBI TGFBR1 TGFBR2 TGFBR3 TGIF1 TGM1 TGM3 TGM5 TGM6 TH THAP1 THBD THBS2 THOC2 THOC6 THPO THRA THRB TIA1 TICAM1 TIMM50 TIMM8A TIMMDC1 TIMP3 TINF2 TJP2 TK2 TKT TLE6 TLK2 TLL1 TLR2 TLR3 TLR5 TM4SF20 TMC1 TMC6 TMC8 TMCO1 TMEM106B TMEM107 TMEM126A TMEM126B TMEM127 TMEM132E TMEM135 TMEM138 TMEM165 TMEM185A TMEM187 TMEM199 TMEM216 TMEM231 TMEM237 TMEM240 TMEM260 TMEM38B TMEM43 TMEM47 TMEM67 TMEM70 TMEM98 TMIE TMLHE TMPO TMPRSS15 TMPRSS3 TMPRSS4 TMPRSS5 TMPRSS6 TMTC3

TMX3 TNC TNF TNFAIP3 TNFRSF10B TNFRSF11A TNFRSF11B TNFRSF13B TNFRSF13C TNFRSF1A TNFRSF4 TNFSF11 TNFSF12 TNFSF4 TNIK TNK2 TNNC1 TNNI2 TNNI3 TNNI3K TNNT1 TNNT2 TNNT3 TNPO2 TNPO3 TNRC6A TNXB TOE1 TOP1 TOP2A TOP3A TOPORS TOR1A TOR1AIP1 TP53 TP53RK TP63 TPH2 TPI1 TPK1 TPM1 TPM2 TPM3 TPM4 TPO TPP1 TPP2 TPRKB TPRN TRAF3 TRAF3IP1 TRAF3IP2 TRAF6 TRAF7 TRAIP TRAK1 TRAP1 TRAPPC11 TRAPPC12 TRAPPC2 TRAPPC6B TRAPPC9 TRDN TREH TREM2 TREX1 TRHR TRIM2 TRIM22 TRIM28 TRIM32 TRIM36 TRIM37 TRIM44 TRIO TRIOBP TRIP11 TRIP12 TRIP13 TRIP4 TRIT1 TRMT1 TRMT10A TRMT10C TRMT5 TRMU TRNT1 TRPA1 TRPC3 TRPC5 TRPC6 TRPM1 TRPM3 TRPM4 TRPM6 TRPM7 TRPS1 TRPV3 TRPV4 TRRAP TSC1 TSC2 TSEN15 TSEN2 TSEN34 TSEN54 TSFM TSGA10 TSHB TSHR TSHZ1 TSPAN12 TSPAN6 TSPAN7 TSPEAR TSPOAP1 TSPYL1 TSPYL2 TSR2 TTBK2 TTC19 TTC21B TTC25 TTC37 TTC7A TTC8 TTI2 TTLL5 TTN TTPA TTR TUB TUBA1A TUBA3D TUBA4A TUBA8 TUBAL3 TUBB TUBB1 TUBB2A TUBB2B TUBB3 TUBB4A TUBB4B TUBB6 TUBB8 TUBG1 TUBGCP4 TUBGCP6 TUFM TULP1 TUSC3 TWIST1 TWIST2 TWNK TXN2 TXNL4A TXNRD2 TYK2 TYMP TYR TYROBP TYRP1 UBA1 UBA5 UBE2A UBE2T UBE3A UBE3B UBIAD1 UBQLN2 UBR1 UBR4 UBR5 UBR7 UBTF UCHL1 UCP1 UCP2 UCP3 UFC1 UFM1 UFSP2 UGT1A1 UMOD UMPS UNC119 UNC13D UNC45B UNC5C UNC80 UNC93B1 UNG UPB1 UPF3B UPK3A UQCC2 UQCC3 UQCRB UQCRC2 UQCRQ UROC1 UROD UROS USB1 USF1 USH1C USH1G USH2A USP11 USP18 USP27X USP8 USP9X USP9Y UTP4 UVSSA VAC14 VAMP1 VANGL1 VANGL2 VAPB VARS1 VARS2 VAX1 VCAN VCL VCP VDAC1 VDR VEGFA VEGFC VHL VIM VIP VIPAS39 VKORC1 VLDLR VMA21 VPS11 VPS13A VPS13B VPS13C VPS13D VPS33A VPS33B VPS35 VPS37A VPS45 VPS4B VPS53 VRK1 VSX1 VSX2 VWA3B VWF WAC WARS1 WARS2 WAS WASHC4 WASHC5 WBP2 WDFY3 WDPCP WDR11 WDR13 WDR19 WDR26 WDR34 WDR35 WDR36 WDR45 WDR45B WDR48 WDR60 WDR62 WDR66 WDR72 WDR73 WDR81 WEE2 WFS1 WHRN WIPF1 WNK1 WNK3 WNK4 WNT1 WNT10A WNT10B WNT2B WNT3 WNT4 WNT5A WNT7A WRAP53 WRN WT1 WWOX XBP1 XDH XIAP XK XPA XPC XPNPEP3 XPO1 XPO5 XPR1 XRCC1 XRCC2 XRCC3 XRCC4 XYLT1 XYLT2 YAP1 YARS1 YARS2 YME1L1 YWHAG YY1 YY1AP1 ZAP70 ZBTB16 ZBTB18 ZBTB20 ZBTB24 ZBTB40 ZBTB42 ZC3H14 ZC4H2 ZCCHC12 ZCCHC8 ZDHHC15 ZDHHC9 ZEB1 ZEB2 ZFAT ZFHX2 ZFHX3 ZFP57 ZFPM2 ZFR ZFYVE26 ZFYVE27 ZIC1 ZIC2 ZIC3 ZMPSTE24 ZMYM3 ZMYM6 ZMYND10 ZMYND11 ZMYND15 ZNF141 ZNF148 ZNF292 ZNF335 ZNF365 ZNF407 ZNF408 ZNF41 ZNF423 ZNF462 ZNF469 ZNF513 ZNF526 ZNF592 ZNF644 ZNF674 ZNF687 ZNF711 ZNF750 ZNF804A ZNF81 ZNHIT3 ZNHIT6 ZP1 ZP2

ZP3 ZRSR2 ZSWIM6.
