# Supplementary material for: A Novel Large Duplication on the X Chromosome as a Cause of Familial Generalized Dystonia: A Case Report
Source: Int J Mol Sci. 2025 Jan 19;26(2):809. doi: 10.3390/ijms26020809 (PMC11765987; doi:10.3390/ijms26020809)
Supplement: Supplementary file 1 [file ijms-26-00809-s001.zip › Supplementary data S2.pdf]

Supplementary Data S2

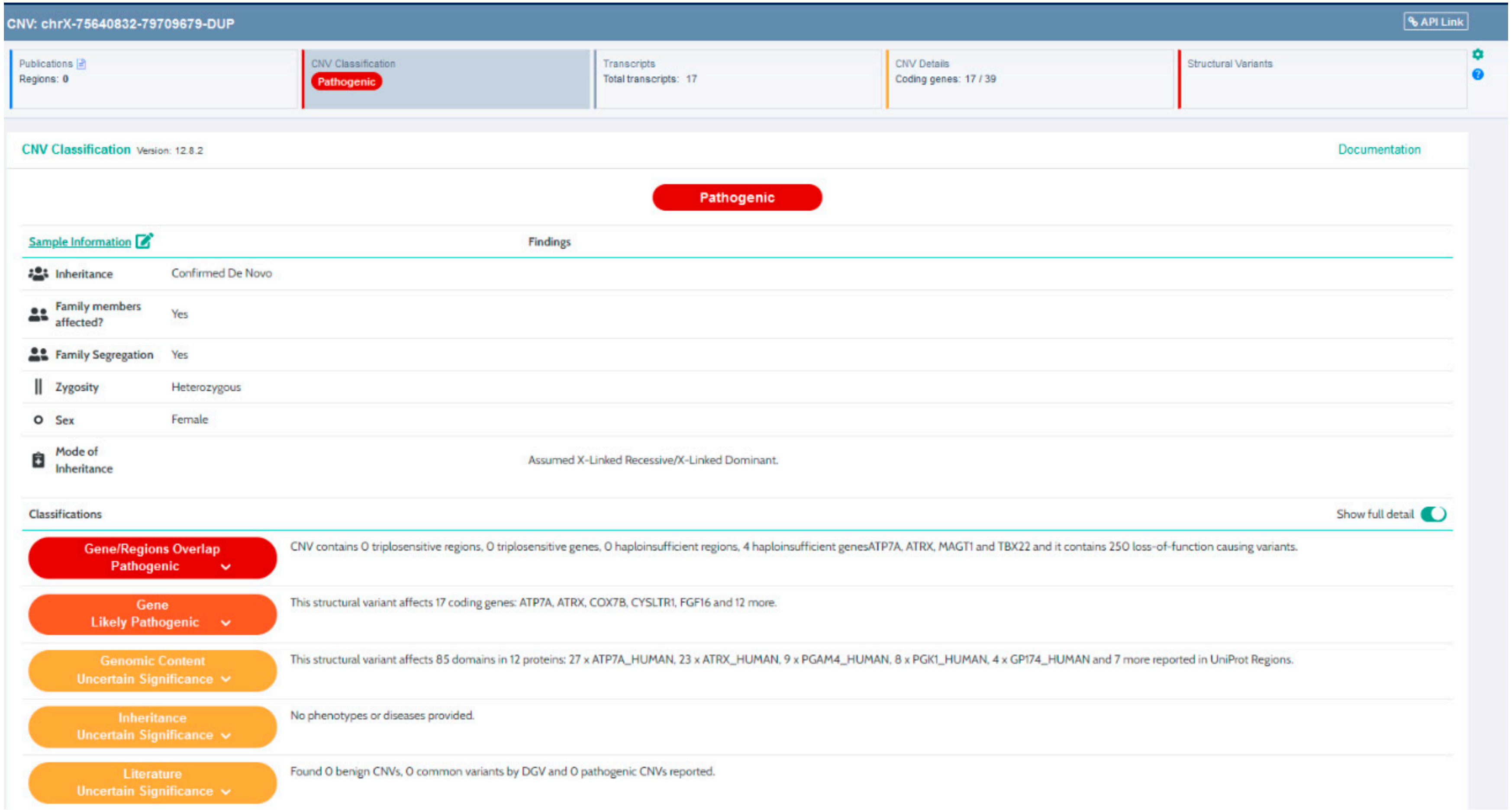

Legend: Classification of the variant chrX:75640831-79709679dup using *Germline Variant Classification* (Version: 12.8.2) from Varsome software (Kopanos et al., 2019 [16]).
